# Resident Development via Progress Testing and Test-Marking: An Innovation and Program Evaluation

**DOI:** 10.7759/cureus.992

**Published:** 2017-01-23

**Authors:** Karen Schiff, D. Josh Williams, Alim Pardhan, Ian Preyra, Shelly-Anne Li, Teresa Chan

**Affiliations:** 1 Division of Emergency Medicine, Department of Medicine, McMaster University; 2 Department of Emergency Medicine, University of British Columbia; 3 Faculty of Nursing, University of Toronto; 4 Faculty of Health Sciences, Division of Emergency Medicine, McMaster University

**Keywords:** progress testing, residency education, program evaluation

## Abstract

**Introduction:**

Since 2008, the McMaster University Royal College Emergency Medicine residency training program has run practice Short Answer Question (SAQ) examinations to help residents test their knowledge and gain practice in answering exam-style questions. However, marking this type of SAQ exam is time-consuming.

**Methods:**

To help address this problem, we require that senior residents help mark at least one exam per year alongside faculty members. Examinees’ identities are kept anonymous by assigning a random number to each resident, which is only decoded after marking. Aggregation of marks is done by faculty only. The senior residents and faculty members all share sequential marking of each question. Each question is reviewed, and exemplar “best practice” answers are discussed. As novel/unusual answers appear, instantaneous fact-checking (via textbooks, or the internet) and discussions occur allowing for real-time modification to the answer keys as needed.

**Results:**

A total of 22 out of 37 residents (post graduate year 1 to post graduate year 5 (PGY1 to PGY5)) participated in a recent program evaluation focus group. This evaluation showed that residents feel quite positive about this process. With the anonymization process, residents do not object to their colleagues seeing and marking their answers. Senior residents have found this process informative and have felt that this process helps them gain insight into better “examsmanship.”

**Conclusions:**

Involving residents in marking short-answer exams is acceptable and perceived as useful experience for improving exam-taking skills. More studies of similar innovations would be required to determine to what extent this may be the case.

## Introduction

### Background

Examinations are still markers of final success for medical learners. Even as we progress to a competency-based framework for postgraduate medical education, it is unlikely that we will entirely abandon this age-old tradition. Additionally, the role of examinations as a part of an assessment portfolio for determining and fostering competence can be better integrated. Testing in medical education has been well-described previously [1-3], with the most recent body of literature focusing on test-enhanced learning (TEL). The theory behind TEL is that well-spaced and frequent testing is beneficial for learning, since each test is a chance for further retrieval practice and theoretically strengthens the connections between concepts [2-3].

Progress testing is a form of formative testing that harnesses the properties of test-enhanced learning and manifests them into a specific practice. As a result, progress testing has been utilized in medical education, both as a method of encouraging retrieval practice and also as a way of allowing learners to gain external insight into their performance compared to their peers, possibly spurring further engagement and interest in studies via social motivation. At McMaster University, we have had a long tradition of frequent progress testing for undergraduate students [4-5], but in postgraduate learning, progress testing is less often used. In most Royal College of Physicians and Surgeons of Canada (RCPSC) Emergency Medicine (EM) residency programs, trainees usually only engage in annual in-training examinations (e.g. the American Board of Emergency Medicine in-training exam, ABEM, or the new Canadian in-training exam, CITE). Few programs engage in additional progress testing, largely because of two factors: 1) Exams are difficult to construct, 2) Exams are also difficult to mark. At McMaster University’s RCPSC EM program, we attempted to create a system that might overcome these barriers and allow our trainees to harness the educational benefits of progress testing and retrieval practice.

### Purpose

Since 2008, McMaster Emergency Medicine has run quarterly, locally-developed progress tests that are aligned with our curricular blocks (dubbed “Block Exams”). The purpose of these progress tests is to encourage continuous studying and review of the curriculum topics and to provide opportunity for retrieval practice. Moreover, these exams consist of short answer questions (SAQs), mirroring the style of both the national in-training exam (CITE) and the terminal examination given by the RCPSC.

Each block, half of our week’s academic time is allocated for a test (75 minutes), usually consisting of seven to 10 SAQs. The answer key is generated by the exam question author and peer reviewed by our associate program director (KS) before use. Questions are aligned to the main topics covered in the preceding three months’ academic time, although there is usually one question per block exam that is meant to force more remote information retrieval.

When this program began in 2009, our program had only 20 residents; however, in the subsequent years our program nearly doubled in size (37 residents at present). As such, in five short years the human resources required to mark and provide good quality feedback to the learners on their practice tests significantly increased.

## Materials and methods

### Description of the innovation

**Figure 1:**
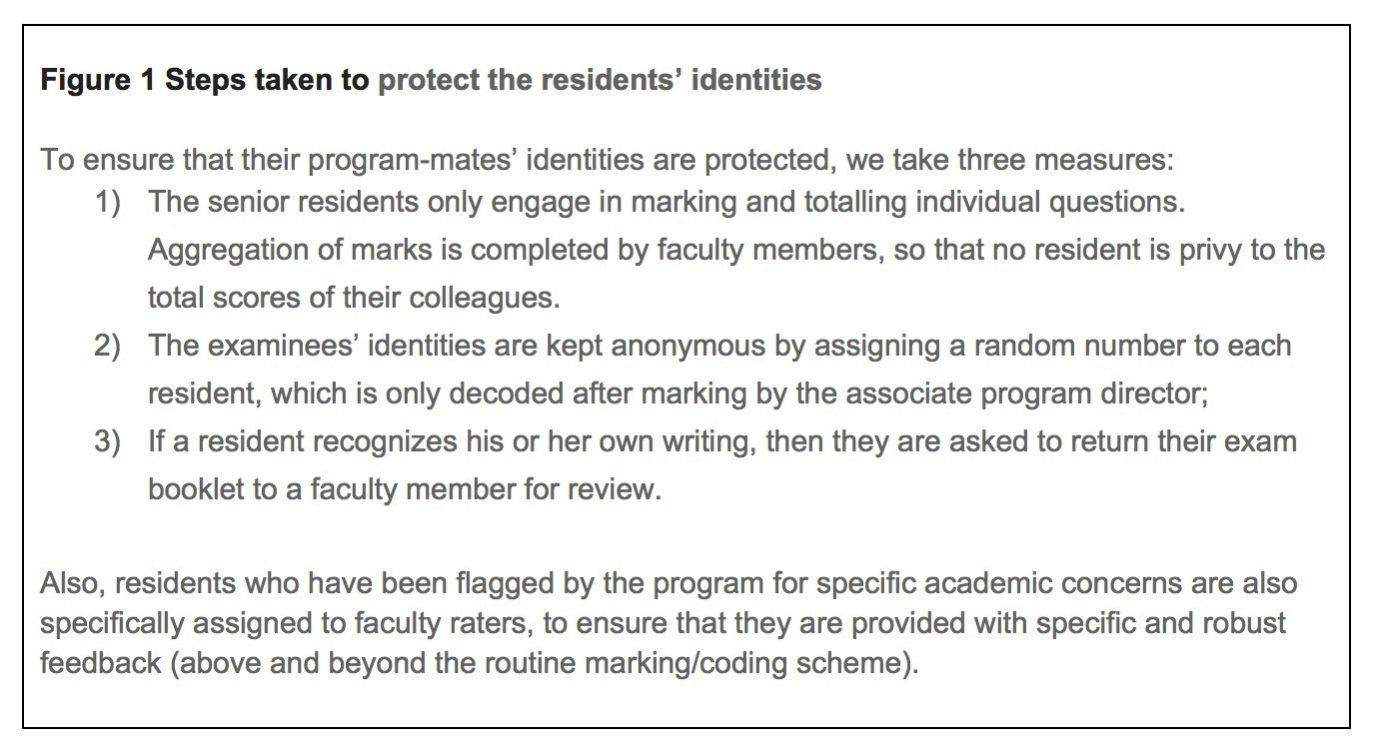
Steps taken to protect the residents’ identities

To help address this problem, our program decided to pilot and then implement a program that involved senior residents in exam marking. Initially this program was volunteer-based; however, as residents participated and saw merit, our program shifted to make this a mandatory experience for all senior residents each year. Currently, we require senior residents to help mark at least one exam per year alongside faculty members. Figure 1 depicts the steps taken to ensure the anonymity of residents’ identities and create a safe procedure for the peer-marking.

### The marking experience

The senior residents and faculty members all share sequential marking of each question. Each question is reviewed and exemplar “best practice” answers are discussed. If novel or unusual answers occur, instantaneous fact-checking (via textbooks, or the internet) and discussions occur allowing for real-time modification to the answer keys as needed.

### Evaluation methods

We conducted a focus group-based program evaluation about this program. We received an exemption from the Hamilton Integrated Research Ethics Board for this program development initiative, and we did receive informed verbal consent from our participants. After receiving an exemption from our institutional review board, we conducted a series of focus groups with our residents using a non-MD (SAL) interviewer. These sessions were recorded and subsequently transcribed and then thematically analyzed with two members of our team (an MD (TC) and a non-MD (SAL)). We used an interpretive descriptive technique [6] for our analysis of these transcripts couched within a realist evaluation framework [7], as we were interested in the pragmatic interaction of our residents and the current educational program. All names were redacted to preserve anonymity.

## Results

Twenty-two out of 37 (60%) residents from our local program participated in our focus groups. Residents were interviewed in their postgraduate year cohorts. Participants ranged from postgraduate year (PGY) 1 to 5. Generally, the progress testing was viewed positively. Our residents noted that the progress testing was beneficial in providing a source of motivation to study and a measure of performance that could be trended overtime and compared to their PGY cohort. Details of the themes, subthemes, codes and quotes that emerged in the focus groups are displayed in Tables 1-2.

Residents discussed the usefulness and barriers for progress testing. The grades from the block exams provided a trend for professional progression in the residency program, which afforded the resident the opportunity for personal reflection on their performance to date. Residents also used grades as a barometer to compare their progress with other peers in the same cohort. The block exams also helped engage residents to identify learning gaps and validate the knowledge and skills acquired during residency. Residents saw block exams as a motivator, specifically for encouraging the review of recently learned content, ensuring performance conformity among their peers, and managing their own identity as a physician in training. Even though residents viewed block exams as a source of stress, they perceived the block exams as a very helpful motivator to review the learned material. Our learners noted variable motivation levels in the various years of residency, with increasing motivation as residents approached their final examinations. Junior residents noted that they were more motivated to learn by clinical encounters and assessments.

We also inquired about their perceptions about the peer marking experience. With the anonymization process, residents did not object to their colleagues seeing and marking their answers. Factors that facilitated acceptability were the use of a standardized marking key and that most residents felt that their identities were protected by the anonymization system (e.g. numbered identifiers). Only one senior resident thought he could identify a peer’s handwriting. One junior resident especially felt that senior residents provided better feedback, since they perceived that these near-peers could better explain how to improve. Furthermore, junior residents perceived senior residents as more reliable and having the ability to provide a more thorough review. Senior residents found that participating as a marker of these exams was informative, many of them remarking that this process helped them gain insight into better exam-taking skills (“examsmanship”). For instance, senior residents felt that having exposure to their peers’ exams during marking would widen their perspective in terms of how certain questions are answered. Overall, the residents agreed that there is educational value in marking exams because it creates an opportunity to discuss and elaborate on the answers with other peers and faculty members.

**Table 1:** Resident perceptions of progress testing

Subtheme	Specifics	Exemplar Quotes*
Provision of formal evaluation (grades)	Specific number on a given test is perceived as not useful	
	Numbers used to trend professional progression	*I like having numbers, I like having numbers over and over again so I can see my trend. – PGY1*
	Barometer to compare performance to peer group	*I think in context it [block exam] is a good barometer for how you are doing – PGY3*
Engages learning	Discovers learning gaps	*I think the exams are motivating…because you need to be shown what you don't know – PGY4*
	Validates learned knowledge and skills	*…[I]t allows you to know what find out what you know and don't know and helps with that piece of sort of your meta-cognitive strategy - Unknown*
	Pushes learners beyond comfort zone	
Provides motivation	Encourages review of recently learned content	*I find that I did try to make a concerted effort with some of the block exams to be kind of like, okay this is a good time to stop and study – PGY2*
	Professional management (motivated to manage their performance as a physician resident)	*I'm motivated to know what is going on when I come to half day because you never know when you are going to get asked a question. So that is a big one for me and I don't want to look like an idiot – PGY2*
	Performance conformity (avoid being an outlier)	*I think that the publishing kind of averages for each year and you don't want to be the one who is significantly lower. So it is a good motivator to keep on studying regularly – PGY4*
	Source of externally precipitated internal motivation	*So while it is meant to be formative and a way of assessing what you know, instead it has turned into a really good motivator to make us study as residents. – PGY4/5*
Identifying resident progress at the systems level	Helps program identify outliers	*I think they [block exams] are more looking for the outliers – PGY2*
	Helps program to begin remediation and assistance of residents-at-risk	*…[T]hose people that are two variations below the mean and need a little bit of a boost up in terms of understanding study strategy – PGY2*
*Exemplar quotes are provided when coherent quote is available.		

Specific	Quotes
Source of Stress	*I know it sucks because we have to study and it's stressful but it is actually very helpful. – PGY2*
Variable use by program director	
Variable motivation (clinical or non-clinical)	
*Exemplar quotes are provided when coherent quote is available.	

 

**Table 2:** Resident perceptions of the peer-marking experience

Theme	Specifics	Exemplar Quotes*
Marking experience	Helped senior residents learn examsmanship	*It is also helpful just to learn, like often they will say there is a better answer sort of learning that gamesmanship of how to best answer your exams and you learn that through marking the block exams. – PGY5*
	Helps to open discussion around best practices for generating answers	*I think there is educational value not only in writing them but in marking them because you actually get to sit around with your colleagues and the staff to discuss the answers, to discuss answers that aren't on the answer key – PGY5*
	Gain empathy for examiners marking exams	
Acceptability	Having senior residents mark block exams were acceptable by most	
	Junior residents perceived having seniors mark exams may be advantageous (near peer effects)	*I think it would be more reliable and because the senior will do a detailed review… - PGY1*
	Standardized marking key increases acceptability	
	Anonymity of tests is important	*They [block exams] are anonymized, so no big deal. It is a good learning experience – PGY5*
*Exemplar quotes are provided when coherent quote is available.		

## Discussion

In the age of competency-based medical education, it is likely that single, final, high-stakes exams may become de-emphasized as we integrate more continuous monitoring of performance. That said, it is likely that there may still be some component of testing in most jurisdictions, as it is simply a very efficient way to ensure knowledge acquisition or retention. The merits of test-enhanced learning have also shown that simply integrating more progress testing may be of benefit for our mid-stream learners who are still developing knowledge and skills.

As such, strategies such as this innovation may allow educators to both more efficiently mentor junior residents through exam-taking skills (i.e. expanding the pool of possible teachers by including senior residents), and also to help prepare senior residents to assume more educational/administrative roles. Not only this, but our program evaluation suggests that substantial adjunctive learning may occur in the senior residents themselves when they are forced to reflect on the answers of others. This may allow senior residents to better calibrate and combat misalignment between self-perceptions of competence and actual efficacy (e.g. the Dunning-Kruger effect) [8]. Indeed, this is aligned with previous work, which has shown that peer assessment of written [9] exams has been useful in various settings. Our findings align with theories of peer learning [10], which may be why junior residents so highly value their senior residents’ feedback.

### Limitations

One of the main limitations of our present study is that the evaluation of our innovation is restricted to a single-centre and a single specialty. We used a constructivist epistemology; however, we are not purporting to attempt to generalize our findings. That said, we feel that the attitudes towards senior-resident development via marking peer resident exams is akin to other literature around near peer mentoring, and hence, we feel that when considering other literature our findings are in alignment with previous work. As such, we feel that there is likely some level of transferability that can be inferred from our findings. Other limitations would be our sample size and convenience sampling. It is possible that we did not reach full data saturation; we did, however, feel our data reached a level of sufficiency [11].

### Future directions

Annually in Canada, the RCPSC EM residents have a short answer question based in-training examination for our specialty. Instead of a centralized marking centre, each residency program is charged with marking their own examinations and then reporting the data. That said, it is often a difficult task to find enough core faculty members who are able to donate their time. Our work suggests there may be opportunity to allow senior residents to mark peer residents' exams during this process, allowing us to expand our evaluation of a peer-based test-marking experience.

## Conclusions

Including residents in the marking of local progress tests can be educationally beneficial for all parties involved. Peer-marking has been deemed to be acceptable or desired by the residents who receive the peer-feedback and therefore should not pose a barrier to implementation. It may be a useful adjunctive educational experience for centers lacking the core faculty to sustain a regular, in-house progress testing system.
